# Effect of Yikou-Sizi powder hot compress on gastrointestinal functional recovery in patients after abdominal surgery: Study protocol for a randomized controlled trial

**DOI:** 10.1097/MD.0000000000012438

**Published:** 2018-09-21

**Authors:** Lixing Cao, Tao Wang, Jinxuan Lin, Zhi Jiang, Qicheng Chen, Huachan Gan, Zhiqiang Chen

**Affiliations:** a The Second Affiliated Hospital of Guangzhou University of Chinese Medicine; b Guangzhou University of Chinese Medicine, Guangzhou, China.

**Keywords:** after abdominal surgery, gastrointestinal functional recovery, randomized controlled trial, Yikou-Sizi powder hot compress

## Abstract

**Background:**

Postoperative gastrointestinal dysfunction (PGD) is a common complication of patients who have undergone surgery. The clinical manifestations cause great discomfort to postoperative patients and can severely affect postoperative recovery. However, although various pharmacologic agents have been explored for several years, success has been limited. Because some commonly used drugs have caused adverse reactions and because abdominal surgery patients generally cannot consume food or medication during the perioperative period, we were prompted to try an external Chinese medicine treatment method. Yikou-Sizi powder hot compress is an efficient therapy in our hospital, but there is a lack of rigorous studies to certify the safety and effectiveness of its external use to improve gastrointestinal motility. This study aimed to introduce the clinical trial design and test the ability of Yikou-Sizi powder hot compress treatment to accelerate gastrointestinal functional recovery after abdominal surgery.

**Methods:**

This study is a randomized controlled clinical trial. The participants will undergo laparoscopic colorectal cancer surgery and laparoscopic total hysterectomy. The primary outcome measure will be the gastrointestinal functional evaluation index, including the time to first passage of flatus, first defecation, first normal bowel sounds, and first consumption of liquid/semigeneral diet foods. According to good clinical practice (GCP), we will evaluate the clinical efficacy and safety of Yikou-Sizi powder hot compress and objectively study the acting mechanism of ghrelin. This pilot trial will be a standard, scientific, and clinical study designed to evaluate the effect of Yikou-Sizi powder hot compress for the recovery of gastrointestinal function after surgery and determine its overall safety.

**Discussion:**

This is the first study to describe the use of Yikou-Sizi powder hot compress to accelerate the recovery of gastrointestinal function after abdominal surgery. The study is designed as a randomized, controlled, clinical, large sample size and pilot trial. Evaluation will consist of combining the primary outcome measures with secondary outcome measures to ensure the objectivity and scientific validity of the study. Due to the observational design and the limited follow-up period, it is not possible to evaluate to what extent the connection between the observed improvement and the interventions represents a causal relationship. Efficient comparison between groups will be analyzed by chi-square test.

## 1 Introduction

Postoperative gastrointestinal dysfunction (PGD) is a common postoperative complication of patients undergoing surgery.^[1–8]^ PGD is caused by surgical trauma, anesthesia, electrolyte disturbance, abdominal cavity inflammation, residual hemorrhage, or drainage tube mechanical stimulation.^[8–10]^ It is characterized by a series of symptoms such as nausea, vomiting, flatulence, defecation difficulty, abdominal distension, and abdominal pain, which are regarded as inevitable reactions after anesthesia and surgery.^[11]^ One of the worst complications is delayed resumption of gastrointestinal function, known as postoperative ileus (POI).^[12]^ This can prolong hospital stays, raise healthcare costs, and increase morbidity and mortality.^[9,13,14]^ POI usually resolves within approximately 3 days, although it can last longer in some cases as a condition termed postoperative paralytic ileus.^[15,16]^ Postoperative use of opioid-based analgesics can increase the incidence of POI.^[17,18]^ The treatment for the recovery of gastrointestinal function mainly focuses on rehydration, oxygen supply, electrolytes, acid–base imbalance, infection prevention, spasticity and pain relief, gastrointestinal decompression, prokinetic drug usage, and related hormonal intervention.^[19]^

Modern medicine attaches great importance to the rapid recovery of gastrointestinal function during the perioperative period, but it lacks active interventional measures. Various pharmacologic agents have been explored for several years, although success has been limited.^[20–28]^ The drugs that have been used as medical treatment for POI are commonly used prokinetic agents such as metoclopramide, domperidone, cisapride, and alvimopan. Domperidone and cisapride can cause adverse reactions that affect the cardiac system,^[29]^ and extrapyramidal reactions exist with metoclopramide use,^[30]^ limiting its clinical application. Its cardiovascular events have brought great risk when used by POI patients.^[31]^ Alvimopan opioid receptor antagonists have recently been developed in the United States for POI, and have been introduced in China, and mainly used in cases of colon failure and to improve gastrointestinal transit function after surgery and decrease postoperative inflammation caused by an excess of nitric oxide that is released after muscular layer infiltration.^[2,32,33]^ Therefore, when the local inflammatory response causes more intense symptoms of POI, alvimopan may no longer be an effective means to promote the recovery of POI.^[33,34]^

It has been proven that Chinese herbal medicines promote the recovery of gastrointestinal function. We have conducted many studies regarding gastrointestinal motility in recent years,^[35–37]^ and there are also many other researchers that have proven that Chinese herbal medicines can promote gastrointestinal functional recovery.^[38–41]^ Although herbal medicine shows some benefit for gastrointestinal motility, abdominal surgery patients generally cannot consume anything by mouth during the perioperative period.

Compared with drugs for internal treatment, the Chinese medicine external treatment method is easy to use, consists of simple materials, causes less adverse reactions, acts quickly, confers a good curative effect that is distinct, results in high patient compliance, and is highly effective. Therefore, Chinese medicine external treatment to promote the recovery of gastrointestinal function after surgery gradually has become a focus in the study of clinical medicine. Hence, rigorous studies are needed to certify the safety and effectiveness of external use of Chinese herbal medicine to improve gastrointestinal motility. Yikou-Sizi powder hot compress is an efficient therapy at our hospital (The Second Affiliated Hospital of Guangzhou University of Chinese Medicine). We consider that anesthesia and surgery lead to POI, which is known as Qi deficiency and Qi stagnation in Chinese medicine syndromes. Yikou-Sizi powder consists of: Fructus Tsaoko (Cao Guo; red cardamom fruit [*Amomum tsaoko*]), Semen Alpiniae Katsumadai (Cao Dou Kou; galangal seeds [*Alpinia katsumadai*]), Fructus Evodiae (Wu Zhu Yu; evodia fruit [*Evodia rutaecarpa*]), and Fructus Alpiniae Oxyphyllae (Yi Zhi Ren; black cardamom fruit [*Alpinia oxyphylla*]). They are all aromatic herbs to enliven and invigorate the spleen and resolve dampness.^[42]^ Furthermore, the 4 drugs (*A tsaoko*,^[43–46]^*A katsumadai*,^[47–55]^*E rutaecarpa*,^[56,57]^ and *A oxyphylla*^[58–60]^) have been studies that have anti-inflammatory, antioxidant activity, neuroprotective, or gastric protective activities. *E rutaecarpa* is effective in the treatment of bowel disorders^[61]^ and evodiamine is the major bioactive compound of *E rutaecarpa* have been shown to increase rat normal jejunal contractility.^[56]^ Both in vitro and in vivo permeation studies showed that *A oxyphylla* essential oils could effectively promote drug absorption across and/or into the skin.^[62]^ Of course, the action of the above mentioned drugs have a positive role to promote gastrointestinal functional recovery.

## 2 Materials and methods

### 2.1 Study design

This study is designed as a randomized, controlled, clinical, pilot trial. We will select patients who underwent laparoscopic colorectal cancer surgery and laparoscopic total hysterectomy between March, 2015 and June, 2019 at Guangdong Provincial Hospital of Chinese Medicine. The patients will be randomly assigned to the Yikou-Sizi powder hot compress group; or the conventional therapy group on the basis of numbers randomly generated using the website (http://www.gztcmgcp.com/sjxt/login.asp). We will evaluate the efficacy and safety of the Yikou-Sizi powder hot compress for patients who have undergone abdominal surgery by comparing the indexes in different groups, such as the time to first passage of flatus, time to first defecation, time to first normal bowel sounds, and time to the initiation of a liquid/semigeneral diet. The study protocol will be certified by the Institutional Review Board (IRB) of The Second Affiliated Hospital of Guangzhou University of Chinese Medicine (Guangdong Provincial Hospital of Chinese Medicine), and registered at the Chinese Clinical Trial Registry (http://www.chictr.org.cn/, ChiCTR-IOR-14005744).

The inclusion criteria, exclusion criteria, and elimination criteria which will be followed to ensure the accuracy of the results are described in the following subsections.

### 2.2 Inclusion criteria

The inclusion criteria are as follows:

1) Underwent laparoscopic colorectal cancer surgery and laparoscopic total hysterectomy
2) Age from 40 to 75 years old
3) Anesthesia time was within 1.5 to 4.5 hours
4) Duration of surgery was 1 to 4 hours
5) The TCM syndrome was identified as Qi stagnation syndrome
6) Willing to sign an informed consent document

### 2.3 Exclusion criteria

The exclusion criteria are as follows:

1) Patients with malignant tumor(s) who required extended radical surgery
2) Advanced malignant tumor with cachexia/extremely weak patient
3) With cardiovascular, liver, kidney, brain, lung, and/or other serious diseases
4) With mental disease
5) Poor control of hypertension, diabetes
6) Pregnant or breastfeeding women
7) Severe malnutrition, serum albumin <21 g/L, prealbumin <0.1 g/L
8) With allergies to the intervention
9) Blood loss of more than 400 mL during surgery
10) Reabdominal surgery and severe intestinal adhesion

### 2.4 Study withdrawal and stopping medication

1) The condition worsens and cannot be controlled in 3 days
2) Does not meet the inclusion criteria, but was included by mistake
3) Refuses to continue the treatment, regardless of the reason
4) Experiences serious complications, infection, or requires a second operation during the treatment
5) Occurrence of other complications because of the interventions, including severe allergy or serious adverse events

### 2.5 Trial procedure

**Figure 1:**
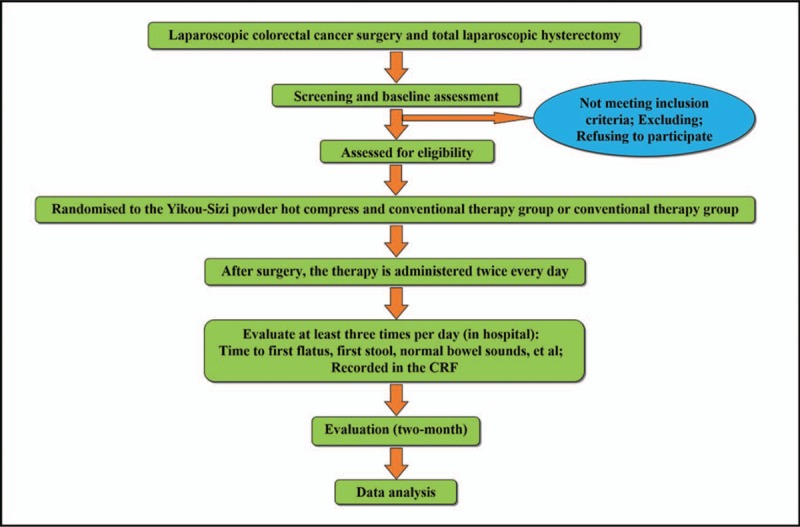
Study flowchart. The entire trial includes a preoperative assessment, an intervention phase (generally 7 days), and a 3-month follow-up phase.

The entire trial includes a preoperative assessment, an intervention phase (generally 7 days), and a 3-month follow-up phase. The included subjects will be randomized to the Yikou-Sizi powder hot compress group or the conventional therapy group through a stratified randomization system, and acquire a random number (corresponding to the Yikou-Sizi powder hot compress and conventional therapy group or conventional therapy group) in this phase. The participants will be instructed to apply a Yikou-Sizi powder hot compress twice every day at 9:00 and 16:00 hours starting after the surgery (the day of surgery was excluded) until the time to defecation. The bowel sounds of the included subjects will be evaluated at least three times per day (1:00–9:00, 9:00–17:00, and 17:00–1:00) after surgery and were recorded in the case report form (CRF) until the time of patient release from the hospital. Patients will be called on the 14th day and the 120th day to inquire about and monitor any adverse events (Fig. 1).

### 2.6 Intervention

The Yikou-Sizi powder will be produced by Guangdong province Engineering Technology Research Institute of TCM, and they hold a Good Manufacturing Practice certificate.

After surgery, the groups will individually receive Yikou-Sizi powder hot compress and conventional therapy or only conventional therapy twice daily at 9:00 and 16:00 hours after surgery until the time to defecation. Conventional therapy will only include antibiotics and liquid support.

### 2.7 Randomization

Participants that had undergone laparoscopic colorectal cancer surgery (n = 220) and laparoscopic total hysterectomy (n = 220) will be randomly assigned to the Yikou-Sizi powder hot compress and conventional therapy group or conventional therapy group. The randomized number will be generated by the network (http://www.gztcmgcp.com/sjxt/login.asp) and saved by statistical professionals.

### 2.8 Quality control

To ensure the quality of the research, this study protocol has undergone multiple modifications and revisions by relevant experienced surgeons, digestive disease specialists, research methodologists, and professional statisticians. To maintain data objectivity, we will ensure that the observers and statisticians were blinded. The entire routine will be monitored by independent quality inspectors. The CRF will be filled in accordance with the CRF instructions; in addition, no alterations will be allowed to be made to the original medical history and data record in the CRF, and any modifications will require a detailed explanation and the signature of the person who made the change. All the laboratory data will be thoroughly recorded, and we repeatedly will check for the existence of any significantly abnormal data and require that the physician notate the records with any necessary instructions regarding them. Because the patients will return any remaining medication, we will record this and be able to objectively determine patient compliance.

### 2.9 Endpoints

Primary endpoints are as follows: time to first flatus, time to first defecation, time to first bowel sounds, time to initiation of a liquid/semiliquid/general diet.

Secondary outcome measures are as follows: gastrointestinal symptoms, such as nausea, abdominal distention and pain, and vomiting; serum levels of hormones, including motilin, ghrelin, bradykinin, and corticotrophin-releasing hormone (CRH); and electrogastrogram.

### 2.10 Safety assessments

The kidney and liver function tests and electrocardiograph will, respectively, be undertaken before and after treatment. Any adverse events will be observed and recorded in detail at any time during the treatment phase, and also the follow-up phase.

### 2.11 Sample size estimation

The sample size estimation formula is based on completely random design sample mean comparison: 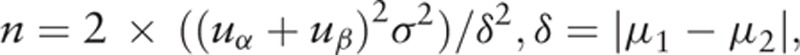


where α = 0.05 and β = 0.2, given a 2-sided test, and n represents the sample size in each group, σ represents the standard deviation, and δ represents the between-group difference with clinical significance. The time to first flatus of the 2 groups based on the preliminary tests and clinical experience is: 
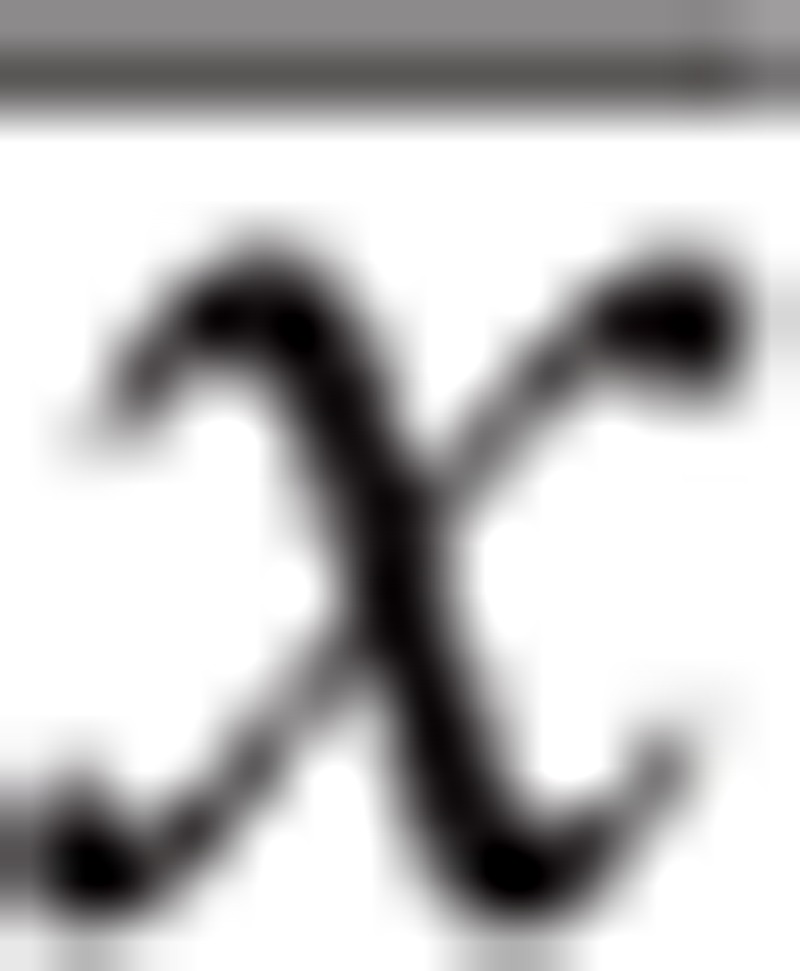
1 = 75.47 hours, S1 = 39.44, 
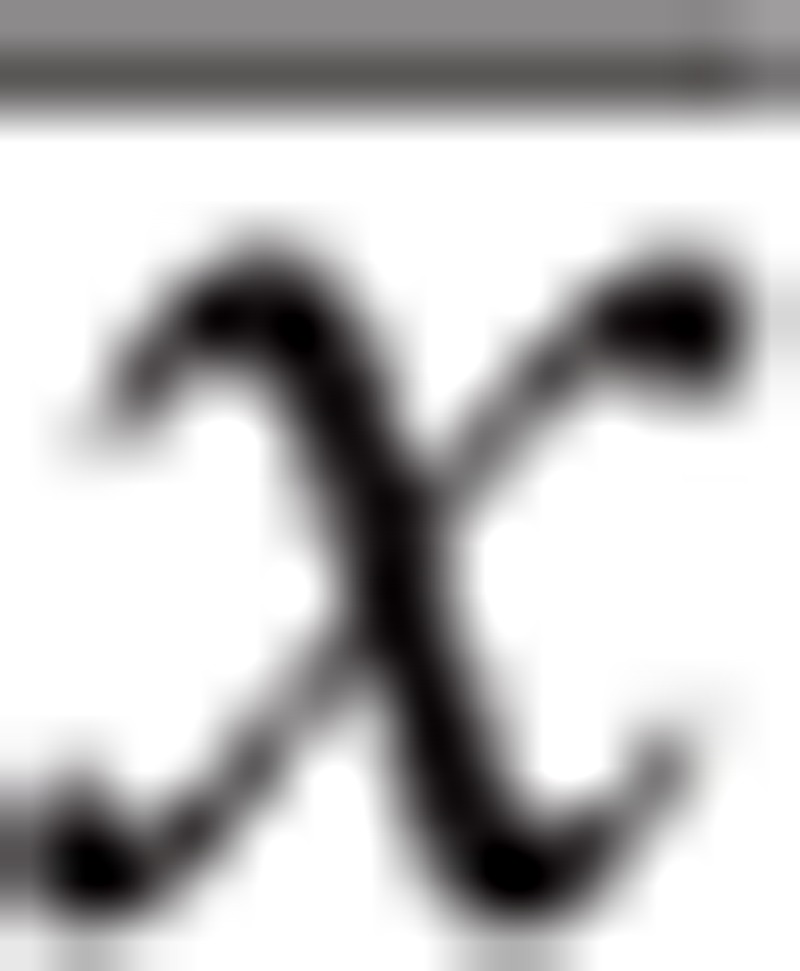
2 = 64.29 hours, S2 = 24.96; the time to bowel sounds is: 
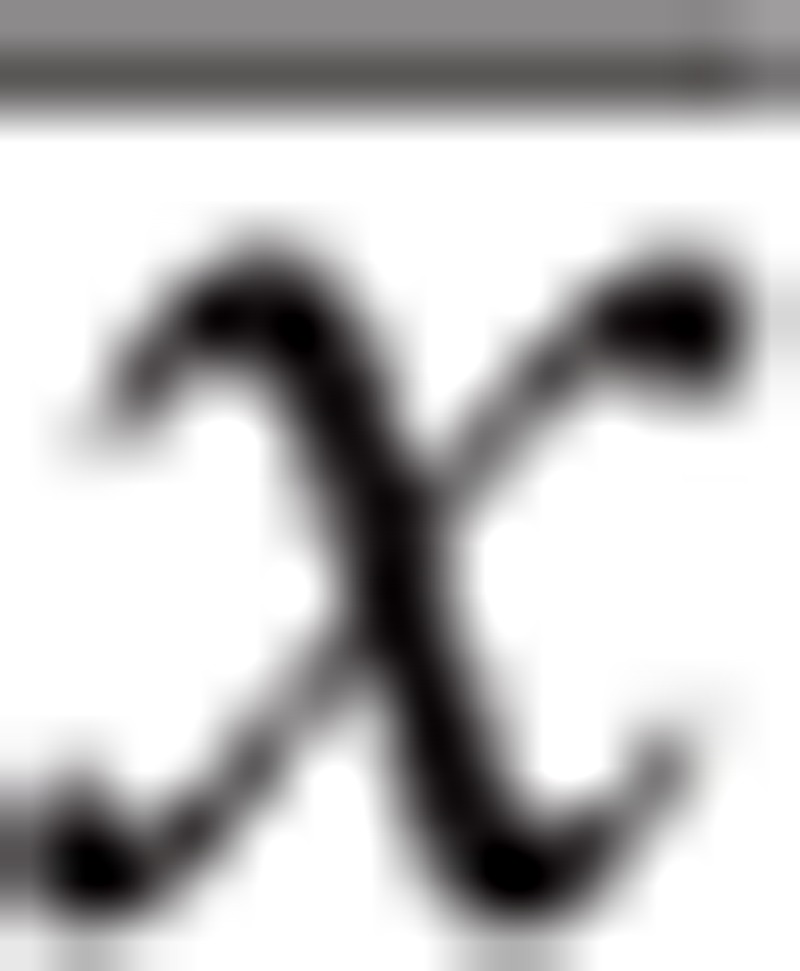
1 = 44.13 hours, S1 = 32.58, 
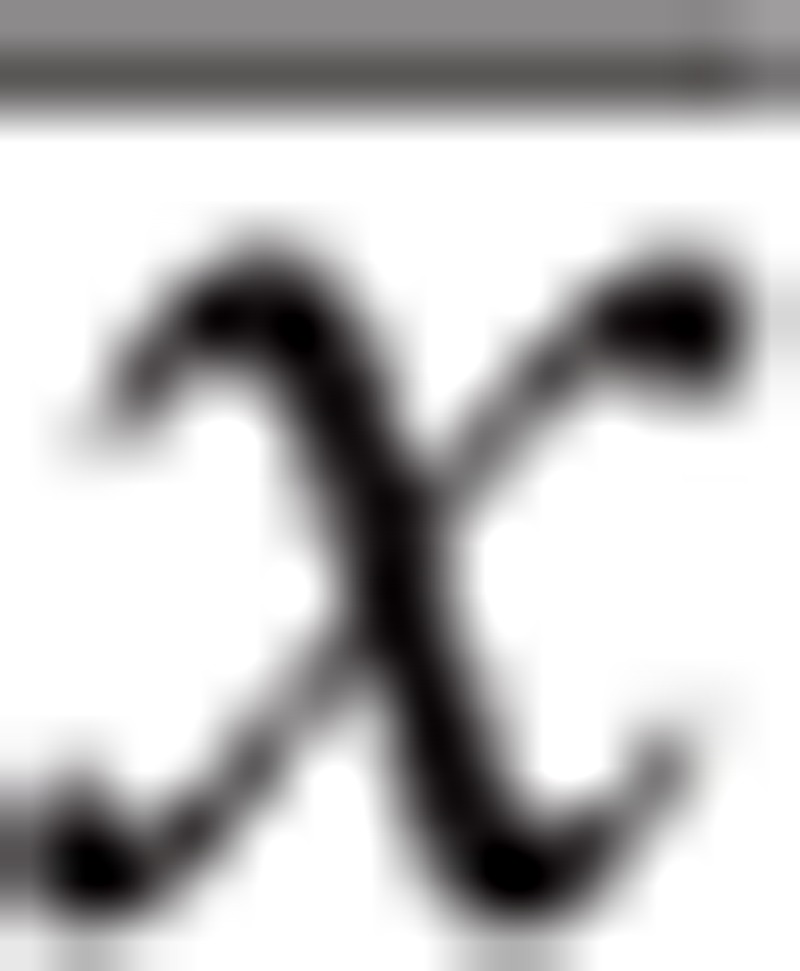
2 = 31.22 hours, S2 = 24.26. The above data were used to estimate the value of the overall parameter. Inserting the data into the PEMS 3.1 for Windows software package, the rounded sample size in each group was 77. Assuming a 15% dropout rate, the final sample size would be 90. The sample size in the groups would be 110 cases. Therefore, the number of participants who have undergone laparoscopic colorectal cancer surgery will be 220, and the number of participants for laparoscopic total hysterectomy will be 220.

### 2.12 Data collection and statistical analysis methods

The data will be collected and recorded in the CRF forms from participants via face-to-face and telephone interviews daily. The Epidata 3.1 Statistical Package was used for data entry. Two people will perform the data entry independently, and they will be trained in advance and must pass a test before they can begin recording the data. The data will then be analyzed using the Statistical Package for the Social Sciences (SPSS) software.

The data will be analyzed by an independent statistician. If the data are divided as measurement data, the mean, standard deviation (SD), minimum value, maximum value, and median will be used for comparison. Comparison among groups will be analyzed by analysis of variance (ANOVA) and pair-wise comparison, while heteroscedasticity data or abnormal distribution will be analyzed by the rank-sum test and pair-wise comparison. If the data are divided as count data, the constituent ratio and frequency will be used for comparison. Efficient comparison between groups will be analyzed by the chi-square test at an α = 0.05 given a 2-sided test.

### 2.13 Ethics approval and consent to participate

The IRB of Guangdong Provincial Hospital of Traditional Chinese Medicine approved the study protocol (approved no. of ethic committee: B2014-058-01). We will obtain verbal informed consent from all study participants.

## 3 Discussion

Treatment of PGD is an important component of perioperative intervention, which has an important influence on the rehabilitation of patients.^[6,8,9]^ The characteristics of TCM syndrome differentiation and treatment, and also a large number of clinical and experimental studies, have confirmed that the application of a Chinese medicine hot compress will accelerate the recovery of gastrointestinal function after surgery.^[35]^ The existing Chinese medicine that is used as an external intervention after abdominal surgery lacks the support of advantageous evidence. There is still a lack of large sample, multicenter, randomized, standardized trials to clinically determine the effectiveness of the Chinese medicine hot compress. Thus, if this therapy is to gain acceptance by evidence-based medicine, more stringent data are required for evaluating the use of the Chinese medicine hot compress to treat PGD.

## 4 Conclusion

This study was designed to evaluate the efficacy and safety of Yikou-Sizi powder hot compress for treating postoperative gastrointestinal dysfunction in abdominal surgery patients, and we wish to provide a scientific basis for therapy efficacy.

## Acknowledgments

We are grateful for the help to all the medical staff of Gastrointestinal Surgery and Gynecology Department in the Second Affiliated Hospital of Guangzhou University of Chinese Medicine.

## Author contributions

**Conceptualization:** Lixing Cao, Tao Wang, Zhiqiang Chen.

**Data curation:** Lixing Cao, Tao Wang.

**Formal analysis:** Lixing Cao, Tao Wang.

**Funding acquisition:** Lixing Cao, Zhiqiang Chen.

**Investigation:** Lixing Cao, Tao Wang, Jinxuan Lin, Zhi Jiang, Qicheng Chen.

**Methodology:** Lixing Cao, Tao Wang, Zhiqiang Chen.

**Project administration:** Lixing Cao, Tao Wang, Huachan Gan.

**Supervision:** Lixing Cao, Tao Wang, Zhiqiang Chen.

**Validation:** Lixing Cao, Tao Wang, Zhiqiang Chen.

**Writing – original draft:** Lixing Cao, Tao Wang, Jinxuan Lin, Zhi Jiang.

**Writing – review & editing:** Lixing Cao, Tao Wang, Qicheng Chen, Huachan Gan.

Zhiqiang Chen orcid: 0000-0001-7658-2746
